# Supplementary material for: A data integration approach unveils a transcriptional signature of type 2 diabetes progression in rat and human islets
Source: PLoS One. 2023 Oct 10;18(10):e0292579. doi: 10.1371/journal.pone.0292579 (PMC10564241; doi:10.1371/journal.pone.0292579)
Supplement: S1 Text — (DOCX) [file pone.0292579.s001.docx]

## Advantages of dual eigen-analysis

## 1. Dual eigen-analysis as an extension to case-control comparison

The dual eigen-analysis, which is the core analytical tool of this study, is a data-driven generalization of the classical two-sample problem. To explain their connection, we performed SVD on profiles of rat islets from one time point of a certain week, and obtained the gene-eigenvector. Meanwhile, we averaged the gene expressions of GK samples and WST samples respectively, and then calculated their expression difference vector. Next, we examined the consistency between the first principal gene-eigenvector and expression difference vector of each time point by their Spearman’s rank correlation coefficient. The correlation coefficients for profiles of all the five time points were close to 1 (Table A below), indicating the gene ranks obtained from SVD and expression differences were highly consistent.

Table A. Spearman’s rank correlation coefficients between the first principal gene-eigenvectors and the expression difference vectors of rat single-time-point data**.**

| **Week** | **Spearman’s correlation** |
| --- | --- |
| 4 | 0.999426 |
| 6 | 0.999001 |
| 8 | 0.996609 |
| 16 | 0.965949 |
| 24 | 0.988596 |

For each single-week time point, we calculated the average expressions of GK samples and WST samples respectively, and then calculated their differences, obtaining the expression difference vector. On the other hand, we calculated the SVD of each single-time-point expression profile, obtaining the gene-eigenvectors. The table shows that the 5 Spearman’s rank correlation coefficients are all close to 1, indicating the gene ranks obtained from SVD and expression differences are highly consistent.

## 2. Superiority of dual eigen-analysis

However, the method of directly comparing gene expression differences cannot well resolve the time-course data due to the heterogeneous samples, whereas the SVD and dual eigen-analysis demonstrated the capability of unveiling the data-driven contrasts of samples and their induced contrasts of genes. Moreover, the dual eigen-analysis of the time-course data has higher detection power, which was demonstrated by the higher significance levels of enriched pathways in comparison to those from the two-sample test using single-time-point data (Fig 3A). It is noted that without prior sample labeling, the dual eigen-analysis was able to distinguish GK islets from WST islets by a contrast of data-driven sample weights (S4 Fig and Fig A below).


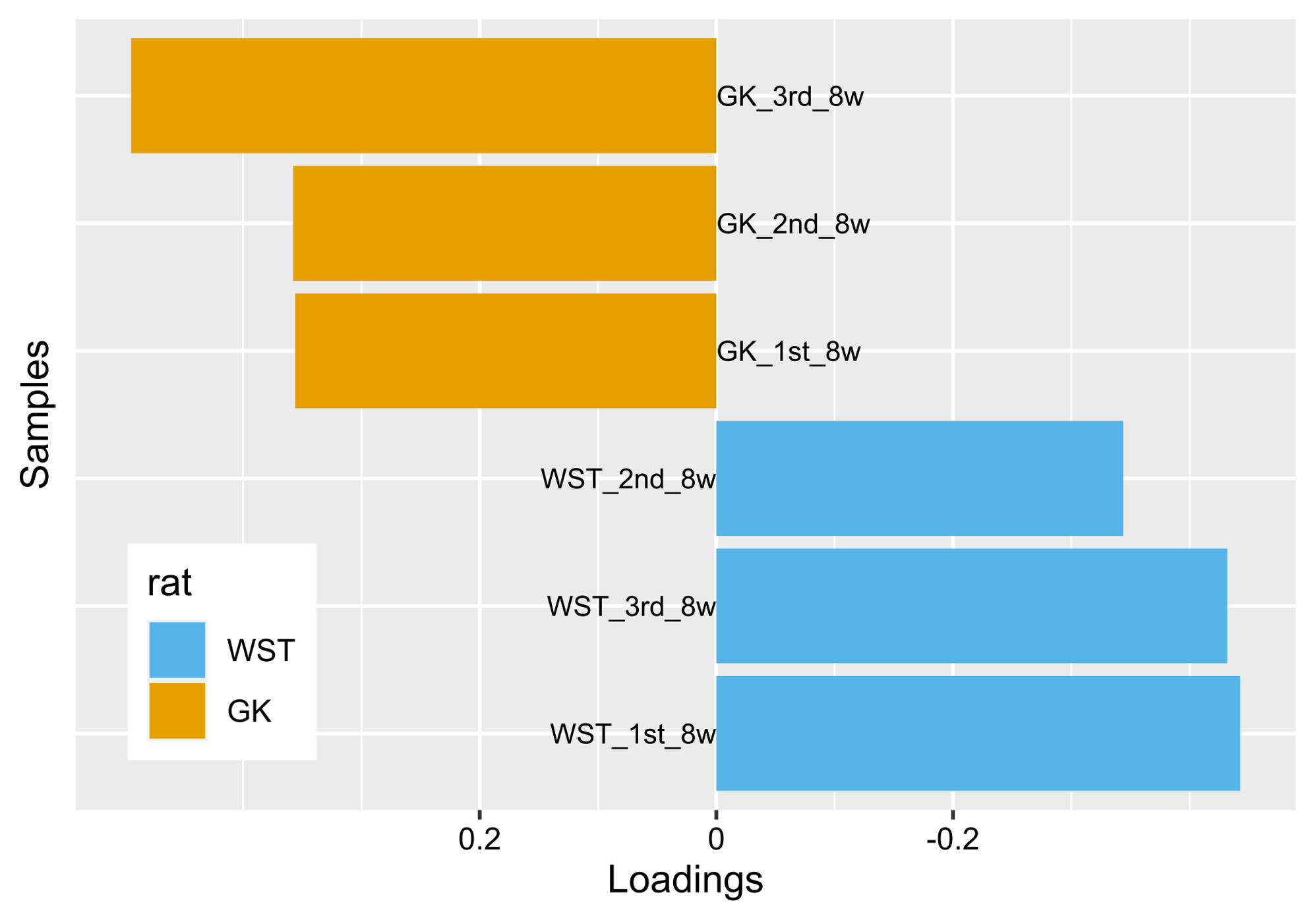


Fig A. Sorted loadings of the first principal sample-eigenvector of rat week-8 data. SVD for the week-8 expression profile of rat islets was calculated. The loadings of GK samples are all positive values, whereas those of WST ones are all negative. GK samples and WST samples are distinguished clearly by their sample loadings.
