# Supplementary material for: A data integration approach unveils a transcriptional signature of type 2 diabetes progression in rat and human islets
Source: PLoS One. 2023 Oct 10;18(10):e0292579. doi: 10.1371/journal.pone.0292579 (PMC10564241; doi:10.1371/journal.pone.0292579)
Supplement: S2 Text — (DOCX) [file pone.0292579.s002.docx]

**The three types of angiogenic genes significantly up-regulated in the aggregated gene-eigenvector of rat and human.**

## 1. Growth factors and their receptors genes

Multiple growth factors regulate the intricate process of vascular development. One such crucial growth factor is VEGF (vascular endothelial growth factor). It enhances EC proliferation and survival, induces EC migration and invasion, and increases the permeability of existing vessels, forming a lattice network for EC migration ([1](#_ENREF_1)). The GO biological process “positive regulation of VEGF production” and the REACTOME pathway “signaling by VEGF” were up-enriched in the angiogenesis gene-eigenvectors of the two species (Fig 2C). Moreover, 6 genes involved in the KEGG “VEGF signaling pathway” were significantly up-regulated in the aggregated gene-eigenvector (S4 Table), and so were 17 genes involved in the REACTOME pathway “signaling by VEGF” (S5 Table).

In addition to VEGF, several other key angiogenic growth factors as well as their receptors were found significantly up-regulated, indicating the activation of their signaling pathways. HGF (hepatocyte growth factor, rank 263, *p*=$6.96\times{10}^{-3}$) is a potent angiogenic molecule and its angiogenic activity is mediated primarily through its direct actions on ECs by binding to its receptor MET (rank 171, *p*=$3.93\times{10}^{-3}$) ([2](#_ENREF_2)). PDGF (platelet-derived growth factor) displays potent biological activities on vascular SMCs, which express abundant PDGF receptors; therefore, it has been generally thought that PDGF directly promotes mural cell recruitment ([3](#_ENREF_3)). Its two isoform genes, *PDGFC* (rank 131, *p*=$2.87\times{10}^{-3}$) and *PDGFD* (rank 393, *p*=0.011), were significantly up-regulated, and so were their two receptor genes, *PDGFRA* (rank 31, *p*=$3.24\times{10}^{-4}$) and *PDGFRB* (rank 575, *p*=0.018). PDGFC is secreted as a latent dimeric factor PDGFCC ([4](#_ENREF_4)), which mobilizes vascular progenitors and promotes their differentiation into ECs and SMCs, stimulates EC migration, and affects SMCs ([5](#_ENREF_5)). PDGFD promotes the angiogenic capacity of endothelial progenitor cells, including proliferation, migration, adhesion and tube formation, and thereby contributes to angiogenesis ([6](#_ENREF_6)). CTGF (connective tissue growth factor, rank 292, *p*=$7.69\times{10}^{-3}$) participates in a variety of direct and indirect mechanisms by which angiogenesis is regulated at multiple control points ([7](#_ENREF_7)). It induces the proliferation, migration, and tube formation of vascular ECs in vitro, and angiogenesis in vivo ([8](#_ENREF_8)).

FGF2 (also known as basic fibroblast growth factor, rank 9, *p*=$7.27\times{10}^{-5}$) is a potent angiogenic factor that can indirectly control neovascularization in concert with other growth factors ([9](#_ENREF_9)). Specifically, FGF2 stimulates VEGF expression in ECs and stromal cells ([10](#_ENREF_10), [11](#_ENREF_11), [12](#_ENREF_12)), and potentiates PDGF-induced vascular maturation possibly through stimulating expression of PDGFR ([13](#_ENREF_13)). FGF2 can also induce HGF expression to drive angiogenesis ([14](#_ENREF_14)). Its two receptor genes *FGFR1* (rank 538, p=0.016) and *FGFR2* (rank 518, *p*=0.015) were both significantly up-regulated in the aggregated gene-eigenvector.

TGF-$\beta$ (transforming growth factor $\beta$) plays a central and indispensable role in angiogenesis ([15](#_ENREF_15), [16](#_ENREF_16), [17](#_ENREF_17)). Its three isoform genes, *TGFB1* (rank 868, *p*=0.031), *TGFB2* (rank 118, *p*=$2.50\times{10}^{-3}$), *TGFB3* (rank 119, *p*=$2.51\times{10}^{-3}$), and the receptor genes, *TGFBR2* (rank 115, *p*=$2.43\times{10}^{-3}$) was significantly up-regulated. TGF-$\beta$ regulates the phase of activation by regulating the activity of positive regulators like FGF2 and VEGF; on the other hand, once a new vessel has formed, TGF-$\beta$1 promotes the phase of resolution by maintaining EC quiescence, inducing vessel maturation, driving basement membrane deposition, and enhancing the interactions between ECs and mural cells ([15](#_ENREF_15), [18](#_ENREF_18), [19](#_ENREF_19)).

## 2. MMP family members genes

Matrix metalloproteinases (MMPs) are a family of zinc-dependent endopeptidases that collectively are capable of degrading essentially all components of the extracellular matrix (ECM) ([20](#_ENREF_20), [21](#_ENREF_21)). At the initial stage of new blood vessel formation, basement membrane, the specialized type IV collagen and laminin-rich connective tissue membrane underlying EC layers, are broken down, so are the interstitial connective tissue matrix, the type I and III collagen-rich ground substances, and the type II collagen in cartilage. In short, angiogenesis requires digestion of the ECM barrier underlying the blood vessel EC layers by MMPs ([22](#_ENREF_22)).

Indeed, we identified multiple MMP family member genes significantly up-regulated in the aggregated gene-eigenvector. Among them, MMP2 (rank 91, *p*=$1.71\times{10}^{-3}$) can cleave both type I and type IV collagens effectively ([22](#_ENREF_22)). MMP14 (rank 321, *p*=$8.75\times{10}^{-3}$) and MMP1 (rank 117 in human, *p*=0.021) are both able to degrade types I, II, and III collagens ([22](#_ENREF_22)). MMP7 (rank 259, *p*=$6.84\times{10}^{-3}$) cleaves collagen type IV and laminin ([23](#_ENREF_23)). It also enhances EC proliferation, up-regulates endothelial expression of MMP1 and MMP2, and induces angiogenesis in vivo ([24](#_ENREF_24), [25](#_ENREF_25)). MMP3 (rank 21 in human, *p*=$3.75\times{10}^{-3}$) and MMP10 (rank 11 in human, *p*=$1.96\times{10}^{-3}$) can both degrade collagen types III, IV, and V, fibronectin, and laminin ([26](#_ENREF_26)). MMP3 can also activate other MMPs such as MMP1 and MMP7 ([27](#_ENREF_27)). Note that MMP1 was exclusively expressed in human, and MMP3 as well as MMP10 did not appear in the rat profile.

## 3. Key EC markers genes

ECs line the blood and lymphatic vessels, forming an interface between the tissues and the blood or lymph. The state of the endothelium is indicated by the phenotype of these cells, represented mainly by (trans) membrane markers. Goncharov et al. ([28](#_ENREF_28)) defined a set of EC markers, among which many were significantly up-regulated in the aggregated gene-eigenvector. Aggregated ranks and p-values of the 23 up-regulated EC marker genes are listed in S3 Table, and 19 of them have supporting evidence for mediating angiogenesis.

Several pro-angiogenic EC markers are highlighted as follows. CD44 (rank 27, *p*=$2.61\times{10}^{-4}$) is a widely expressed cell surface adhesion molecule promoting pathological angiogenesis through its regulation of diverse functions of ECs, such as proliferation, migration, adhesion, invasion, and communication with the microenvironment ([29](#_ENREF_29)). VCAM1 (rank 4, *p*=$1.53\times{10}^{-5}$), which binds to integrin $\alpha_{4}\beta_{1}$, promotes close intercellular adhesion between ECs and pericytes and this interaction is required for blood vessel formation ([30](#_ENREF_30)). CD93 (rank 128, *p*=$2.85\times{10}^{-3}$) is required for tubular morphogenesis, migration, and adhesion of ECs, and plays a key role in organizing the endothelial cytoskeleton and cell junctions ([31](#_ENREF_31)). KLF4 (rank 268, *p*=$7.03\times{10}^{-3}$) is a transcription factor, which is a central regulator of sprouting angiogenesis via regulating Notch signaling pathway ([32](#_ENREF_32)). TIE2 (rank 1119, *p*=0.040) is an essential receptor for vascular maturation during developmental, physiological and pathological angiogenesis ([33](#_ENREF_33)). ADAM9 (rank 352, *p*=$9.85\times{10}^{-3}$) enhances angiogenesis by increasing the shedding of several angiogenesis-related endothelial membrane proteins including TIE2, VEGFR2, and VCAM1 ([34](#_ENREF_34)).

**References**

1. Niu G, Chen X. Vascular endothelial growth factor as an anti-angiogenic target for cancer therapy. Curr Drug Targets. 2010;11(8):1000-17.

2. Bussolino F, Di Renzo MF, Ziche M, Bocchietto E, Olivero M, Naldini L, et al. Hepatocyte growth factor is a potent angiogenic factor which stimulates endothelial cell motility and growth. J Cell Biol. 1992;119(3):629-41.

3. Ostman A. PDGF receptors-mediators of autocrine tumor growth and regulators of tumor vasculature and stroma. Cytokine Growth Factor Rev. 2004;15(4):275-86.

4. Fredriksson L, Li H, Fieber C, Li X, Eriksson U. Tissue plasminogen activator is a potent activator of PDGF-CC. The EMBO journal. 2004;23(19):3793-802.

5. Li X, Tjwa M, Moons L, Fons P, Noel A, Ny A, et al. Revascularization of ischemic tissues by PDGF-CC via effects on endothelial cells and their progenitors. J Clin Invest. 2005;115(1):118-27.

6. Zhang J, Zhang H, Chen Y, Fu J, Lei Y, Sun J, et al. Platelet‑derived growth factor D promotes the angiogenic capacity of endothelial progenitor cells. Mol Med Rep. 2019;19(1):125-32.

7. Brigstock DR. Regulation of angiogenesis and endothelial cell function by connective tissue growth factor (CTGF) and cysteine-rich 61 (CYR61). Angiogenesis. 2002;5(3):153-65.

8. Shimo T, Nakanishi T, Nishida T, Asano M, Kanyama M, Kuboki T, et al. Connective tissue growth factor induces the proliferation, migration, and tube formation of vascular endothelial cells in vitro, and angiogenesis in vivo. J Biochem. 1999;126(1):137-45.

9. Murakami M, Simons M. Fibroblast growth factor regulation of neovascularization. Curr Opin Hematol. 2008;15(3):215-20.

10. Seghezzi G, Patel S, Ren CJ, Gualandris A, Pintucci G, Robbins ES, et al. Fibroblast growth factor-2 (FGF-2) induces vascular endothelial growth factor (VEGF) expression in the endothelial cells of forming capillaries: an autocrine mechanism contributing to angiogenesis. J Cell Biol. 1998;141(7):1659-73.

11. Claffey KP, Abrams K, Shih SC, Brown LF, Mullen A, Keough M. Fibroblast growth factor 2 activation of stromal cell vascular endothelial growth factor expression and angiogenesis. Lab Invest. 2001;81(1):61-75.

12. Tsunoda S, Nakamura T, Sakurai H, Saiki I. Fibroblast growth factor-2-induced host stroma reaction during initial tumor growth promotes progression of mouse melanoma via vascular endothelial growth factor A-dependent neovascularization. Cancer Sci. 2007;98(4):541-8.

13. Nissen LJ, Cao R, Hedlund EM, Wang Z, Zhao X, Wetterskog D, et al. Angiogenic factors FGF2 and PDGF-BB synergistically promote murine tumor neovascularization and metastasis. J Clin Invest. 2007;117(10):2766-77.

14. Onimaru M, Yonemitsu Y, Tanii M, Nakagawa K, Masaki I, Okano S, et al. Fibroblast growth factor-2 gene transfer can stimulate hepatocyte growth factor expression irrespective of hypoxia-mediated downregulation in ischemic limbs. Circ Res. 2002;91(10):923-30.

15. Pepper MS. Transforming growth factor-beta: vasculogenesis, angiogenesis, and vessel wall integrity. Cytokine Growth Factor Rev. 1997;8(1):21-43.

16. Dickson MC, Martin JS, Cousins FM, Kulkarni AB, Karlsson S, Akhurst RJ. Defective haematopoiesis and vasculogenesis in transforming growth factor-beta 1 knock out mice. Development. 1995;121(6):1845-54.

17. Oshima M, Oshima H, Taketo MM. TGF-beta receptor type II deficiency results in defects of yolk sac hematopoiesis and vasculogenesis. Dev Biol. 1996;179(1):297-302.

18. Marchuk DA, Srinivasan S, Squire TL, Zawistowski JS. Vascular morphogenesis: tales of two syndromes. Hum Mol Genet. 2003;12 Spec No 1:R97-112.

19. Azhar M, Schultz Jel J, Grupp I, Dorn GW, 2nd, Meneton P, Molin DG, et al. Transforming growth factor beta in cardiovascular development and function. Cytokine Growth Factor Rev. 2003;14(5):391-407.

20. Birkedal-Hansen H, Moore WG, Bodden MK, Windsor LJ, Birkedal-Hansen B, DeCarlo A, et al. Matrix metalloproteinases: a review. Crit Rev Oral Biol Med. 1993;4(2):197-250.

21. Woessner JF, Jr. The family of matrix metalloproteinases. Ann N Y Acad Sci. 1994;732:11-21.

22. Sang QX. Complex role of matrix metalloproteinases in angiogenesis. Cell Res. 1998;8(3):171-7.

23. Sbardella D, Fasciglione GF, Gioia M, Ciaccio C, Tundo GR, Marini S, et al. Human matrix metalloproteinases: an ubiquitarian class of enzymes involved in several pathological processes. Mol Aspects Med. 2012;33(2):119-208.

24. Huo N, Ichikawa Y, Kamiyama M, Ishikawa T, Hamaguchi Y, Hasegawa S, et al. MMP-7 (matrilysin) accelerated growth of human umbilical vein endothelial cells. Cancer Lett. 2002;177(1):95-100.

25. Nishizuka I, Ichikawa Y, Ishikawa T, Kamiyama M, Hasegawa S, Momiyama N, et al. Matrilysin stimulates DNA synthesis of cultured vascular endothelial cells and induces angiogenesis in vivo. Cancer Lett. 2001;173(2):175-82.

26. Laronha H, Caldeira J. Structure and Function of Human Matrix Metalloproteinases. Cells. 2020;9(5).

27. Verma RP, Hansch C. Matrix metalloproteinases (MMPs): chemical-biological functions and (Q)SARs. Bioorg Med Chem. 2007;15(6):2223-68.

28. Goncharov NV, Nadeev AD, Jenkins RO, Avdonin PV. Markers and Biomarkers of Endothelium: When Something Is Rotten in the State. Oxid Med Cell Longev. 2017;2017:9759735.

29. Chen L, Fu C, Zhang Q, He C, Zhang F, Wei Q. The role of CD44 in pathological angiogenesis. Faseb j. 2020;34(10):13125-39.

30. Garmy-Susini B, Jin H, Zhu Y, Sung RJ, Hwang R, Varner J. Integrin alpha4beta1-VCAM-1-mediated adhesion between endothelial and mural cells is required for blood vessel maturation. J Clin Invest. 2005;115(6):1542-51.

31. Langenkamp E, Zhang L, Lugano R, Huang H, Elhassan TE, Georganaki M, et al. Elevated expression of the C-type lectin CD93 in the glioblastoma vasculature regulates cytoskeletal rearrangements that enhance vessel function and reduce host survival. Cancer Res. 2015;75(21):4504-16.

32. Hale AT, Tian H, Anih E, Recio FO, 3rd, Shatat MA, Johnson T, et al. Endothelial Kruppel-like factor 4 regulates angiogenesis and the Notch signaling pathway. J Biol Chem. 2014;289(17):12016-28.

33. Fagiani E, Christofori G. Angiopoietins in angiogenesis. Cancer Lett. 2013;328(1):18-26.

34. Guaiquil V, Swendeman S, Yoshida T, Chavala S, Campochiaro PA, Blobel CP. ADAM9 is involved in pathological retinal neovascularization. Mol Cell Biol. 2009;29(10):2694-703.
