# Supplementary material for: A data integration approach unveils a transcriptional signature of type 2 diabetes progression in rat and human islets
Source: PLoS One. 2023 Oct 10;18(10):e0292579. doi: 10.1371/journal.pone.0292579 (PMC10564241; doi:10.1371/journal.pone.0292579)
Supplement: S3 Text — (DOCX) [file pone.0292579.s003.docx]

## Metabolism switch in the gene signature

The up-regulated LDHA (lactate dehydrogenase A, rank 254, *p*=$6.67\times{10}^{-3}$) preferentially catalyzes the conversion of pyruvate to lactate, and the conversion of NADH to NAD+ in anaerobic conditions, or fermentation ([1](#_ENREF_1)). Two enzyme genes *GOT1* (rank -350, *p*=0.011) and *MDH1* (rank -300, *p*=$9.03\times{10}^{-3}$) were enriched at the opposite pole to angiogenesis, namely the same pole as oxidative phosphorylation in the angiogenesis gene-eigenvectors, implying the malate-aspartate shuttle (Fig A below) were defective in the type 2 diabetic islets. A similar down-regulation pattern was previously reported in GK islets ([2](#_ENREF_2)). Such a defect could increase the cytosolic NADH/NAD+ ratio, enhance the cytoplasmic fermentation, and disrupt the mitochondrial metabolism ([3](#_ENREF_3)). This metabolism switch was consistent with the down-enrichment of pathways involved in oxidative phosphorylation as observed in Fig 2C.

Fig A. Metabolism map of malate-aspartate shuttle and cytoplasmic fermentation. It was reconstructed by adapting a figure from a reference article ([2](#_ENREF_2)) with minor modifications.

**References**

1. Read JA, Winter VJ, Eszes CM, Sessions RB, Brady RL. Structural basis for altered activity of M- and H-isozyme forms of human lactate dehydrogenase. Proteins. 2001;43(2):175-85.

2. Hou J, Li Z, Zhong W, Hao Q, Lei L, Wang L, et al. Temporal Transcriptomic and Proteomic Landscapes of Deteriorating Pancreatic Islets in Type 2 Diabetic Rats. Diabetes. 2017;66(8):2188-200.

3. Eto K, Tsubamoto Y, Terauchi Y, Sugiyama T, Kishimoto T, Takahashi N, et al. Role of NADH shuttle system in glucose-induced activation of mitochondrial metabolism and insulin secretion. Science. 1999;283(5404):981-5.
