# Supplementary material for: A data integration approach unveils a transcriptional signature of type 2 diabetes progression in rat and human islets
Source: PLoS One. 2023 Oct 10;18(10):e0292579. doi: 10.1371/journal.pone.0292579 (PMC10564241; doi:10.1371/journal.pone.0292579)
Supplement: S4 Text — (DOCX) [file pone.0292579.s004.docx]

## Protective mechanisms of decorin and PEDF

Decorin and PEDF have protective effects against diabetes complications in multiple organs and tissues. Specifically, the expression of decorin was increased during the development of diabetic kidney disease and decorin deficiency enhanced progressive nephropathy in diabetic mice ([1](#_ENREF_1)). Decorin could ameliorate diabetic macular edema development by preventing retinal pigment epithelial barrier disruption induced by high glucose plus hypoxia through suppression of p38 mitogen-activated protein kinase (MAPK) activation ([2](#_ENREF_2)). In diabetic cardiomyopathy, decorin overexpression inhibited the fibrosis and inflammation through reduction of TGF-$\beta$1 and inactivation of NF-$\kappa$B pathway ([3](#_ENREF_3)). Similarly, elevated circulating PEDF levels were previously observed in diabetic retinopathy, nephropathy, and coronary artery diseases, which might represent a protective counter-regulatory mechanism ([4](#_ENREF_4), [5](#_ENREF_5), [6](#_ENREF_6)).

**References**

1. Williams KJ, Qiu G, Usui HK, Dunn SR, McCue P, Bottinger E, et al. Decorin deficiency enhances progressive nephropathy in diabetic mice. Am J Pathol. 2007;171(5):1441-50.

2. Wang S, Du S, Wu Q, Hu J, Li T. Decorin Prevents Retinal Pigment Epithelial Barrier Breakdown Under Diabetic Conditions by Suppressing p38 MAPK Activation. Invest Ophthalmol Vis Sci. 2015;56(5):2971-9.

3. Chen F, Lai J, Zhu Y, He M, Hou H, Wang J, et al. Cardioprotective Effect of Decorin in Type 2 Diabetes. Front Endocrinol (Lausanne). 2020;11:479258.

4. Ogata N, Matsuoka M, Matsuyama K, Shima C, Tajika A, Nishiyama T, et al. Plasma concentration of pigment epithelium-derived factor in patients with diabetic retinopathy. J Clin Endocrinol Metab. 2007;92(3):1176-9.

5. Hui E, Yeung CY, Lee PC, Woo YC, Fong CH, Chow WS, et al. Elevated circulating pigment epithelium-derived factor predicts the progression of diabetic nephropathy in patients with type 2 diabetes. J Clin Endocrinol Metab. 2014;99(11):E2169-77.

6. Wang F, Ma X, Zhou M, Pan X, Ni J, Gao M, et al. Serum pigment epithelium-derived factor levels are independently correlated with the presence of coronary artery disease. Cardiovasc Diabetol. 2013;12:56.
