# Supplementary material for: A data integration approach unveils a transcriptional signature of type 2 diabetes progression in rat and human islets
Source: PLoS One. 2023 Oct 10;18(10):e0292579. doi: 10.1371/journal.pone.0292579 (PMC10564241; doi:10.1371/journal.pone.0292579)
Supplement: S1 Fig — (PDF) [file pone.0292579.s005.pdf]

Figure S1

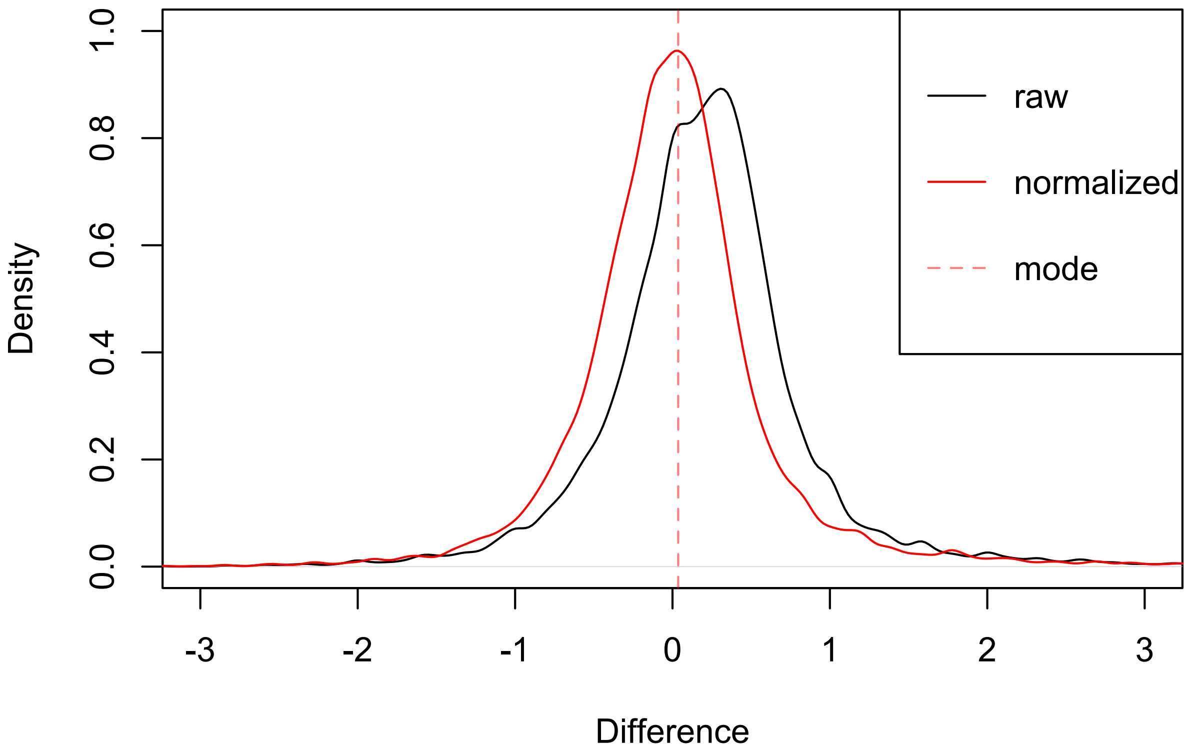

Figure S1. Densities of pairwise differences between rat sample 1 and sample 2 before and after normalization by MUREN. The mode of the density of pairwise differences is transformed to near zero after normalization.
