# Supplementary material for: A data integration approach unveils a transcriptional signature of type 2 diabetes progression in rat and human islets
Source: PLoS One. 2023 Oct 10;18(10):e0292579. doi: 10.1371/journal.pone.0292579 (PMC10564241; doi:10.1371/journal.pone.0292579)
Supplement: S2 Fig — (PDF) [file pone.0292579.s006.pdf]

Figure S2

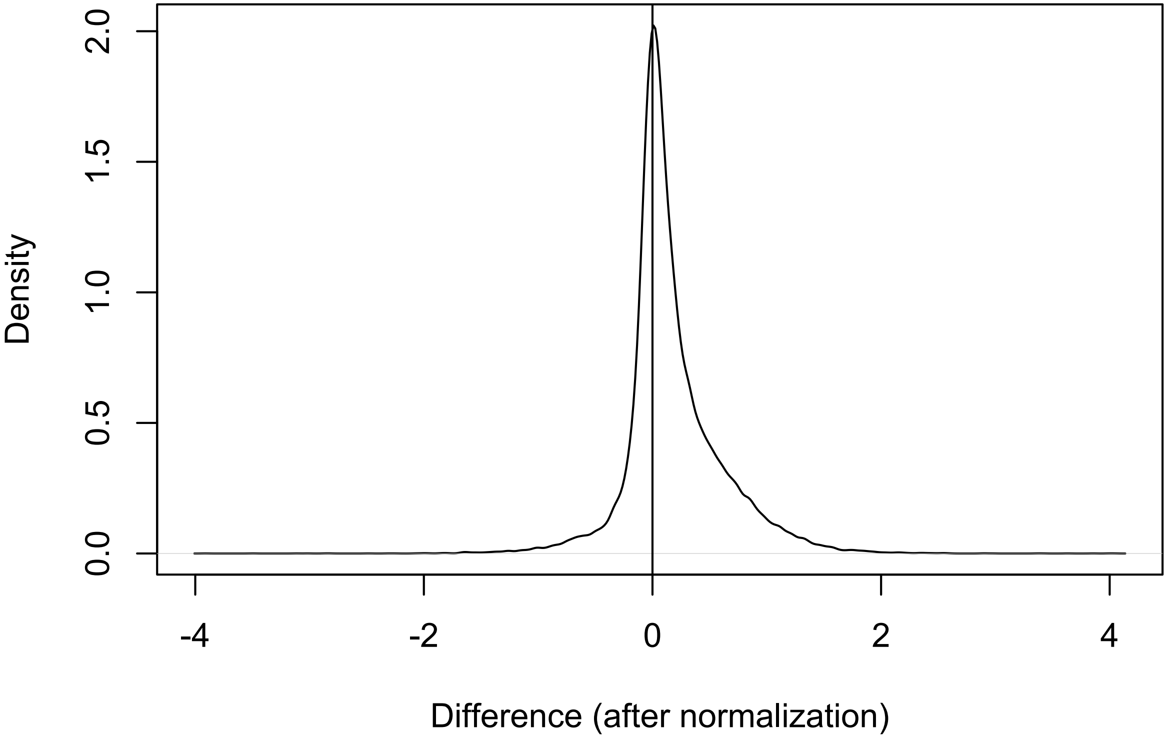

Figure S2. Density of pairwise differences between human samples GSM946735 and GSM946750 after sub-sub normalization. The mode of the density is almost at zero.
