# Supplementary material for: A data integration approach unveils a transcriptional signature of type 2 diabetes progression in rat and human islets
Source: PLoS One. 2023 Oct 10;18(10):e0292579. doi: 10.1371/journal.pone.0292579 (PMC10564241; doi:10.1371/journal.pone.0292579)
Supplement: S3 Fig — (PDF) [file pone.0292579.s007.pdf]

Figure S3

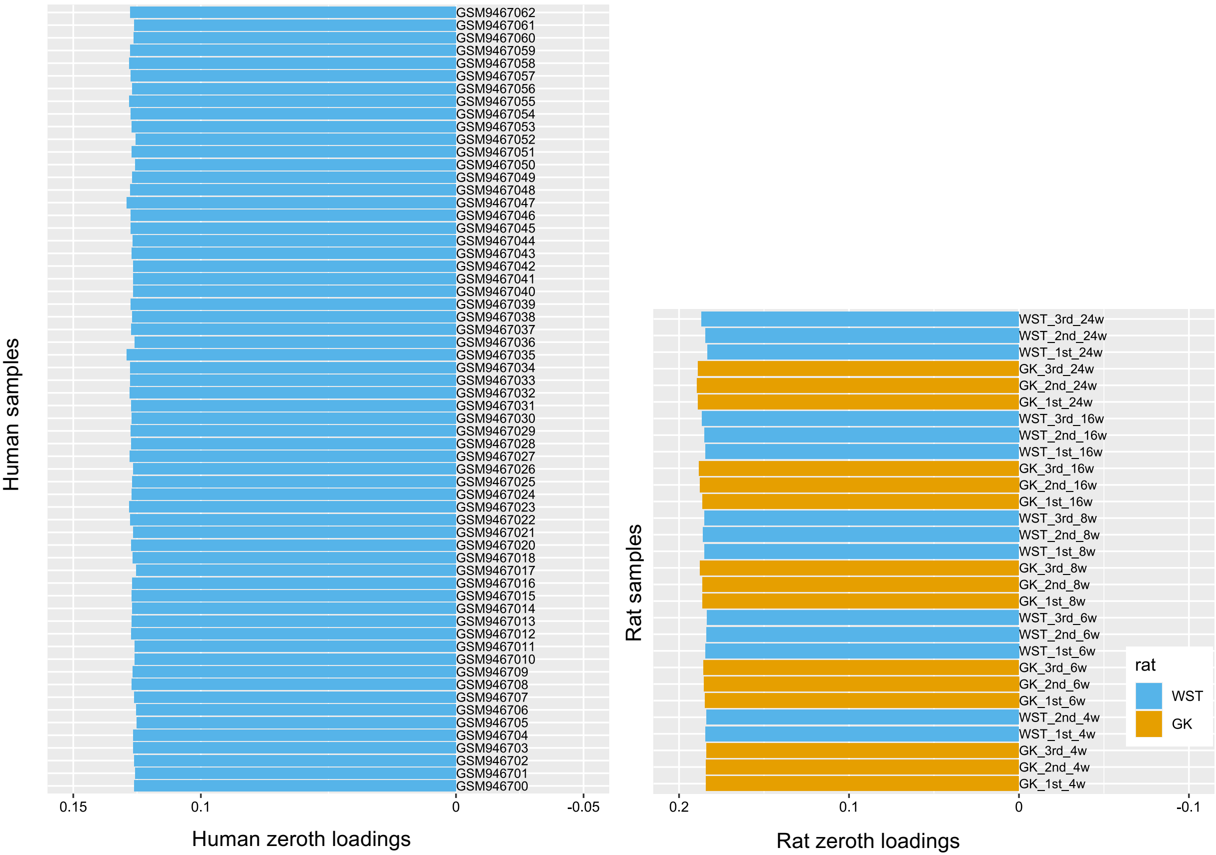

Figure S3. Loadings of the zeroth principal sample-eigenvector of rat and human. The loadings are almost identical. The loadings of human are around $1/\sqrt{62} \approx 0.127$, and the coefficient of variation (CV) is 0.64%. The loadings of rat are around $1/\sqrt{29} \approx 0.186$, and the CV is 0.44%.
