# Supplementary material for: A data integration approach unveils a transcriptional signature of type 2 diabetes progression in rat and human islets
Source: PLoS One. 2023 Oct 10;18(10):e0292579. doi: 10.1371/journal.pone.0292579 (PMC10564241; doi:10.1371/journal.pone.0292579)
Supplement: S4 Fig — (PDF) [file pone.0292579.s008.pdf]

Figure S4

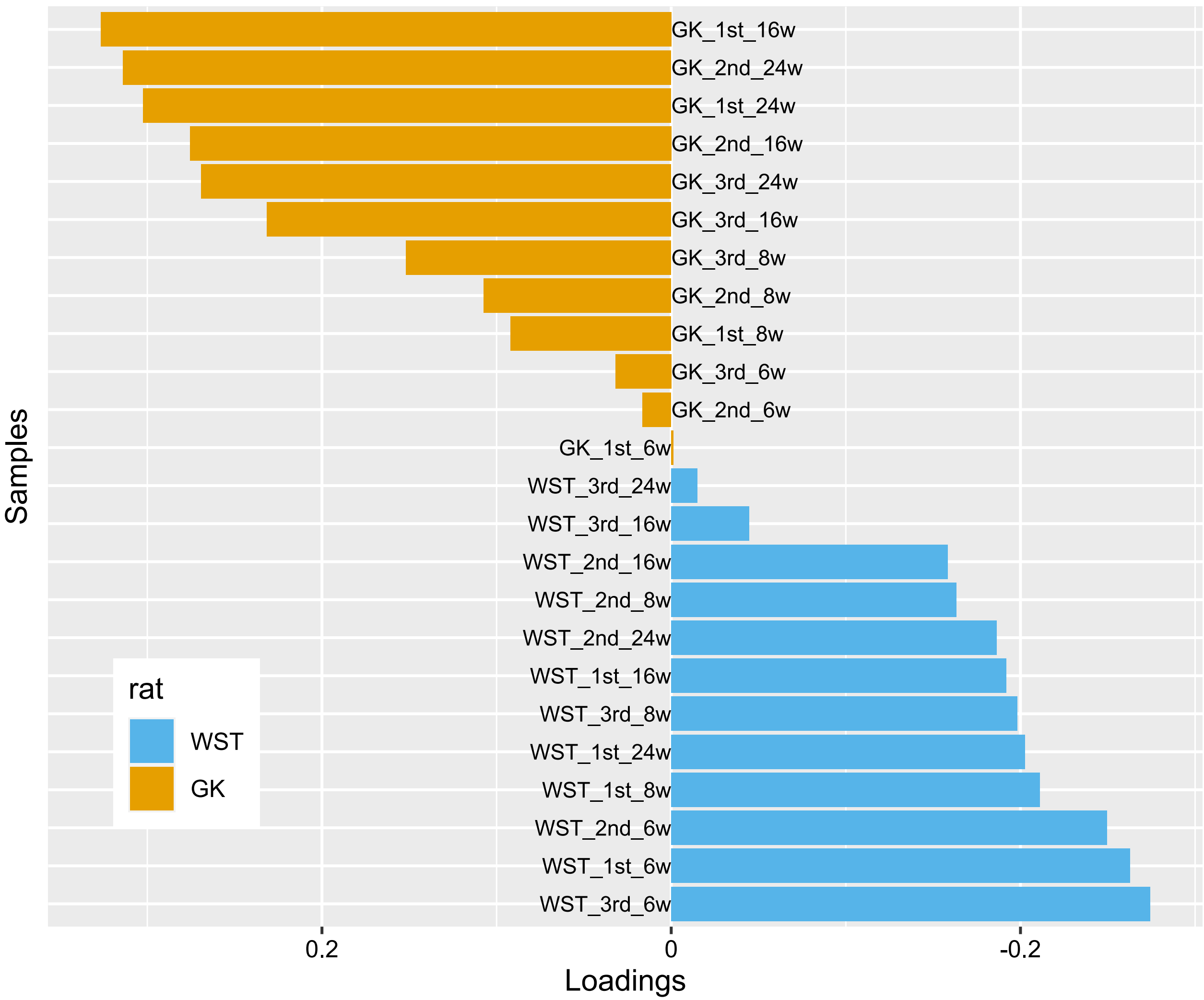

Figure S4. Sorted loadings of the first principal sample-eigenvector of rat data excluding week 4. Rats at week 4 are still in the development stage, and are thus subject to different regulation from that of mature rats. We excluded week-4 data from the original expression profiles of rat islets, and then carried out SVD. The first principal loadings of GK samples are almost positive values, whereas those of WST ones are all negative. GK and WST samples are perfectly separated by their loadings.
