# Supplementary material for: A data integration approach unveils a transcriptional signature of type 2 diabetes progression in rat and human islets
Source: PLoS One. 2023 Oct 10;18(10):e0292579. doi: 10.1371/journal.pone.0292579 (PMC10564241; doi:10.1371/journal.pone.0292579)
Supplement: S5 Fig — (PDF) [file pone.0292579.s009.pdf]

Figure S5

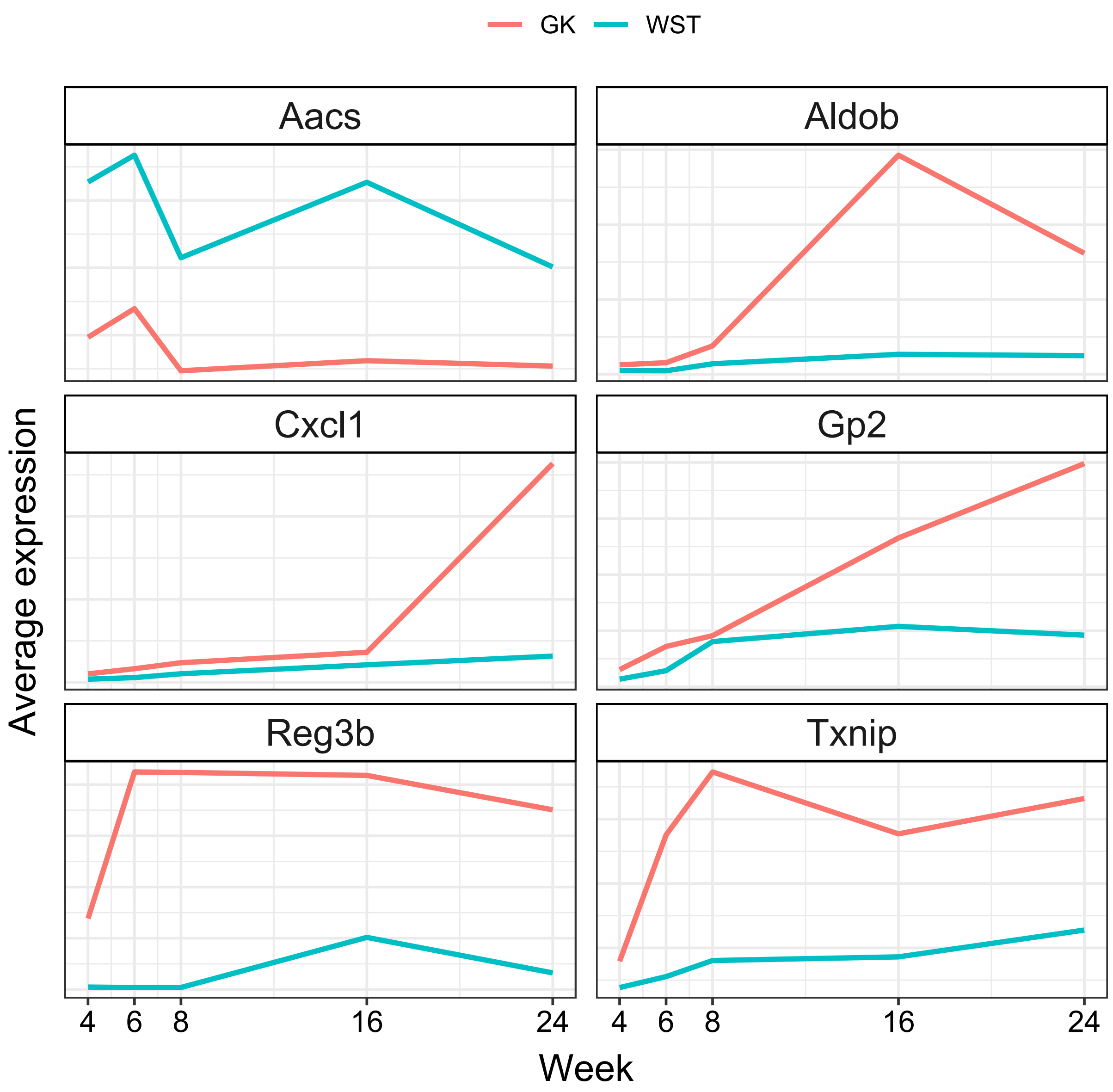

Figure S5. Consistency between the time-course expressions of GK and WST islets by RNA-seq and RT-PCR data. The averages of the three replicates were shown for the RNA-seq data of 6 genes over time. These 6 genes were randomly selected for the validation of the RNA-seq data by the qRT-PCR methods. Figure S3(C) in (1) shows the qRT-PCR data of the 6 genes at weeks 4, 8 and 16.

References

1. Hou J, Li Z, Zhong W, Hao Q, Lei L, Wang L, et al. Temporal Transcriptomic and Proteomic Landscapes of Deteriorating Pancreatic Islets in Type 2 Diabetic Rats. Diabetes. 2017;66(8):2188-200.
