# Supplementary material for: A data integration approach unveils a transcriptional signature of type 2 diabetes progression in rat and human islets
Source: PLoS One. 2023 Oct 10;18(10):e0292579. doi: 10.1371/journal.pone.0292579 (PMC10564241; doi:10.1371/journal.pone.0292579)
Supplement: S6 Fig — (PDF) [file pone.0292579.s010.pdf]

Figure S6

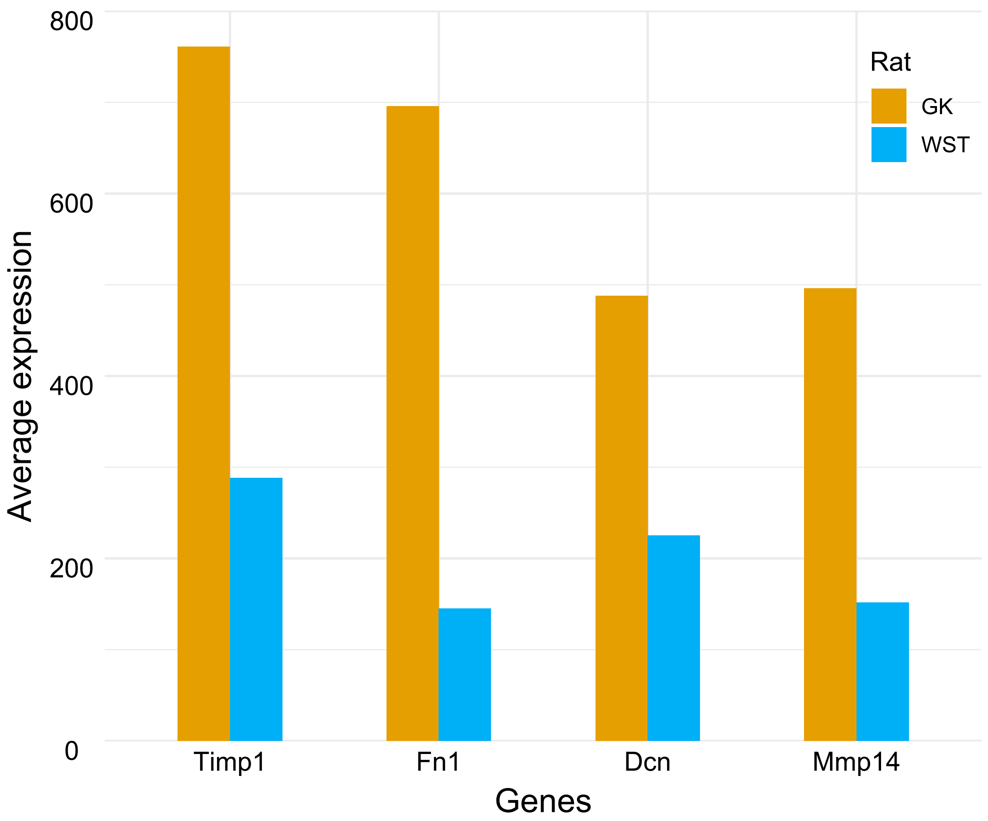

Gene symbols	*Timp1*	*Fn1*	*Dcn*	*Mmp14*
Fold changes	5.6	11	2.2	6.2

Figure S6. Consistency between the time-course expressions of GK and WST islets by RNA-seq and RT-PCR data of the 4-month-old rats from another study. The study in (1) obtained the mRNA expressions of several genes by RT-PCR. We selected 4 genes involved in angiogenesis. At the bottom are the RT-PCR fold changes of these 4 genes. Then we consider the rats of 16 weeks old from the time-course expression profiles. The bar graphs show the average reads of 3 GK islets and of 3 WST islets. The results from the two expression data were quite consistent.

References

1. Homo-Delarche F, Calderari S, Irminger JC, Gangnerau MN, Coulaud J, Rickenbach K, et al. Islet inflammation and fibrosis in a spontaneous model of type 2 diabetes, the GK rat. Diabetes. 2006;55(6):1625-33.
