# Supplementary material for: A data integration approach unveils a transcriptional signature of type 2 diabetes progression in rat and human islets
Source: PLoS One. 2023 Oct 10;18(10):e0292579. doi: 10.1371/journal.pone.0292579 (PMC10564241; doi:10.1371/journal.pone.0292579)
Supplement: S7 Fig — (PDF) [file pone.0292579.s011.pdf]

Figure S7

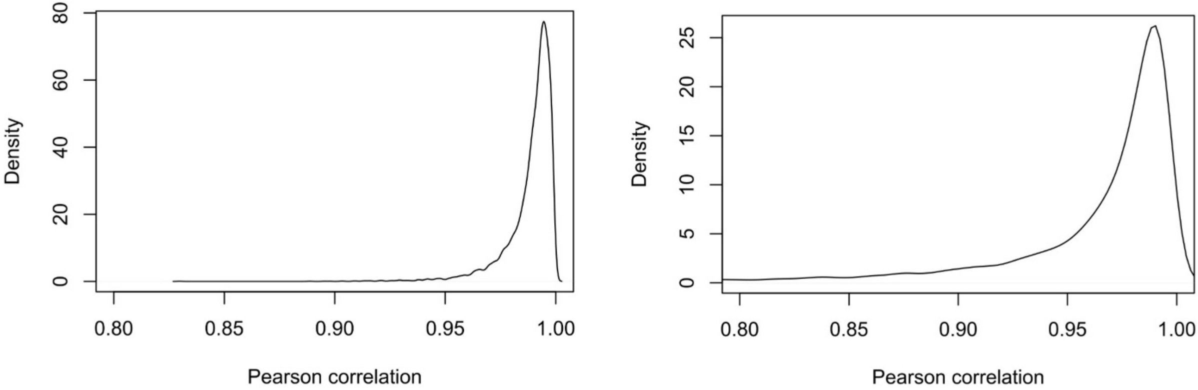

Figure S7. The stability of the human top two gene-eigenvectors by re-sampling islets. The Pearson correlation coefficients are concentrated between 0.95 and 1.
