# Supplementary material for: A data integration approach unveils a transcriptional signature of type 2 diabetes progression in rat and human islets
Source: PLoS One. 2023 Oct 10;18(10):e0292579. doi: 10.1371/journal.pone.0292579 (PMC10564241; doi:10.1371/journal.pone.0292579)
Supplement: S8 Fig — (PDF) [file pone.0292579.s012.pdf]

Figure S8

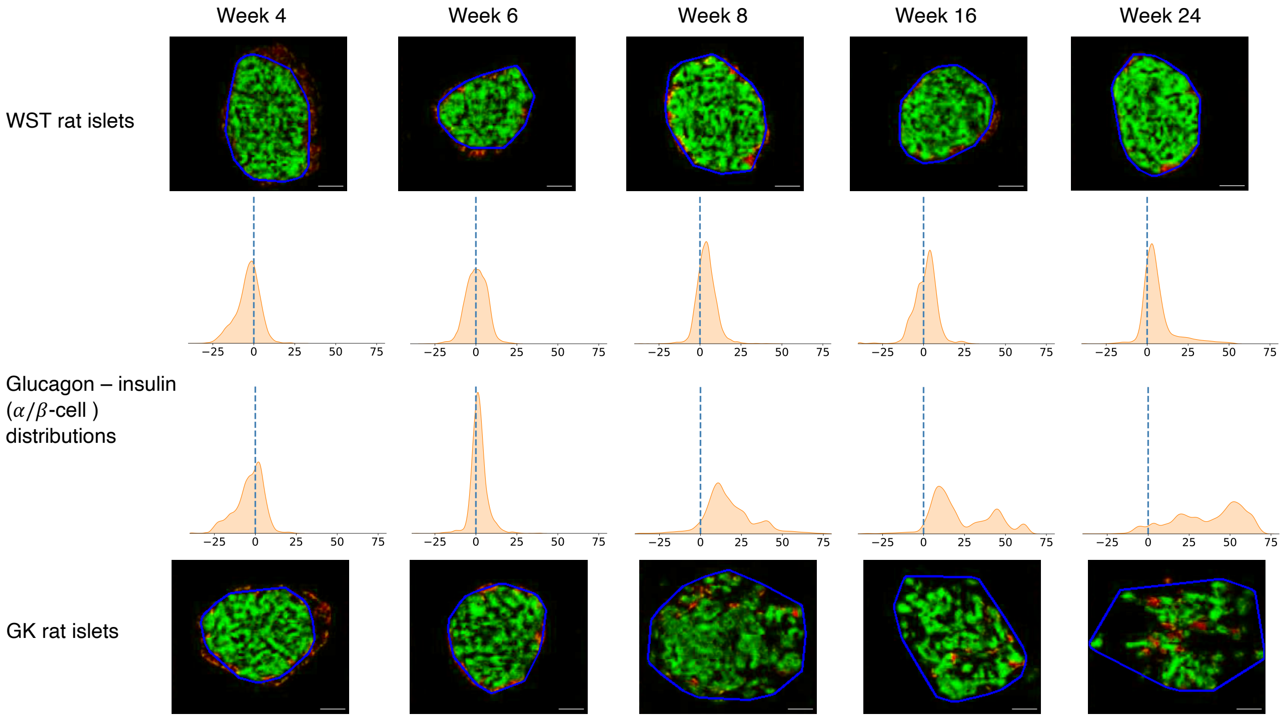

Figure S8. Quantification of pancreatic α- and β-cells spatial distribution along the T2D progression. In each rat islet micrograph (scale bar shown at the bottom is 50 μm), the rim (blue line) of the insulin region (green) is sketched, and the distances of glucagon pixels (red) away from the rim are defined. Pixels inside the rim have positive distances and those outside have negative ones. A kernel density of the distances is shown next to each micrograph. It quantifies the spatial distribution of α- and β-cells via that of glucagon and insulin. The spatial distributions of GK islets in the early stage (4-6 weeks) are similar to those of WST islets, which are approximately around zero. Distributions of GK islets in the late stage (8-24 weeks) shift to the right progressively and become flatter, indicating that α-cells gradually penetrate into islets.
