# Supplementary material for: A data integration approach unveils a transcriptional signature of type 2 diabetes progression in rat and human islets
Source: PLoS One. 2023 Oct 10;18(10):e0292579. doi: 10.1371/journal.pone.0292579 (PMC10564241; doi:10.1371/journal.pone.0292579)
Supplement: S9 Fig — (PDF) [file pone.0292579.s013.pdf]

Figure S9

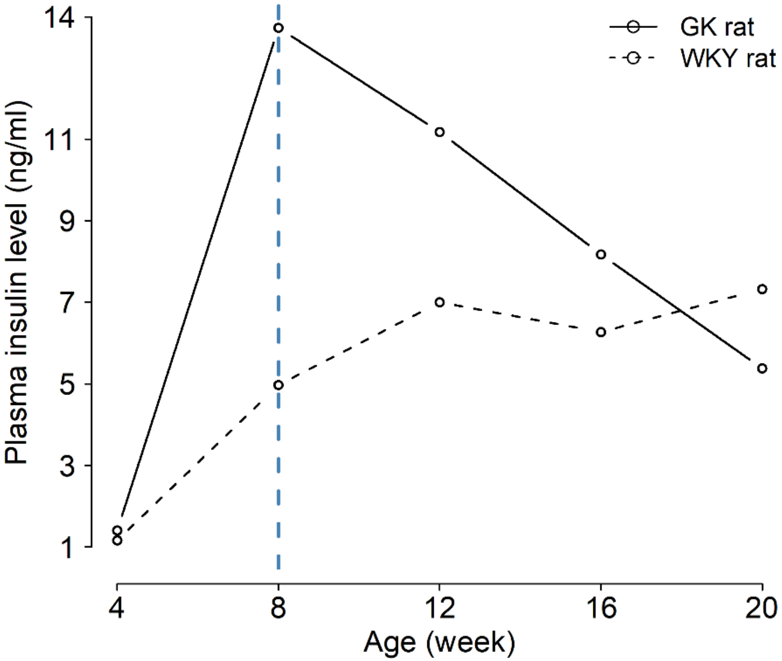

Figure S9. The plasma insulin levels of GK and WKY rats over time. In the study (1), the plasma insulin levels were measured for GK and Wistar-Kyoto (WKY, a substrain of Wistar) rats at ages: 4, 8, 12, 16, and 20 weeks. The averages of six rats at each time point were shown. The pattern of an up-trend before week 8 and a down-trend after week 8 observed in GK rats is similar to that of insulin mRNA levels in Fig 3E-F.

References

1. Gao W, Bihorel S, DuBois DC, Almon RR, Jusko WJ. Mechanism-based disease progression modeling of type 2 diabetes in Goto-Kakizaki rats. J Pharmacokinet Pharmacodyn. 2011;38(1):143-62.
