# Supplementary material for: A data integration approach unveils a transcriptional signature of type 2 diabetes progression in rat and human islets
Source: PLoS One. 2023 Oct 10;18(10):e0292579. doi: 10.1371/journal.pone.0292579 (PMC10564241; doi:10.1371/journal.pone.0292579)
Supplement: S10 Fig — (PDF) [file pone.0292579.s014.pdf]

Figure S10

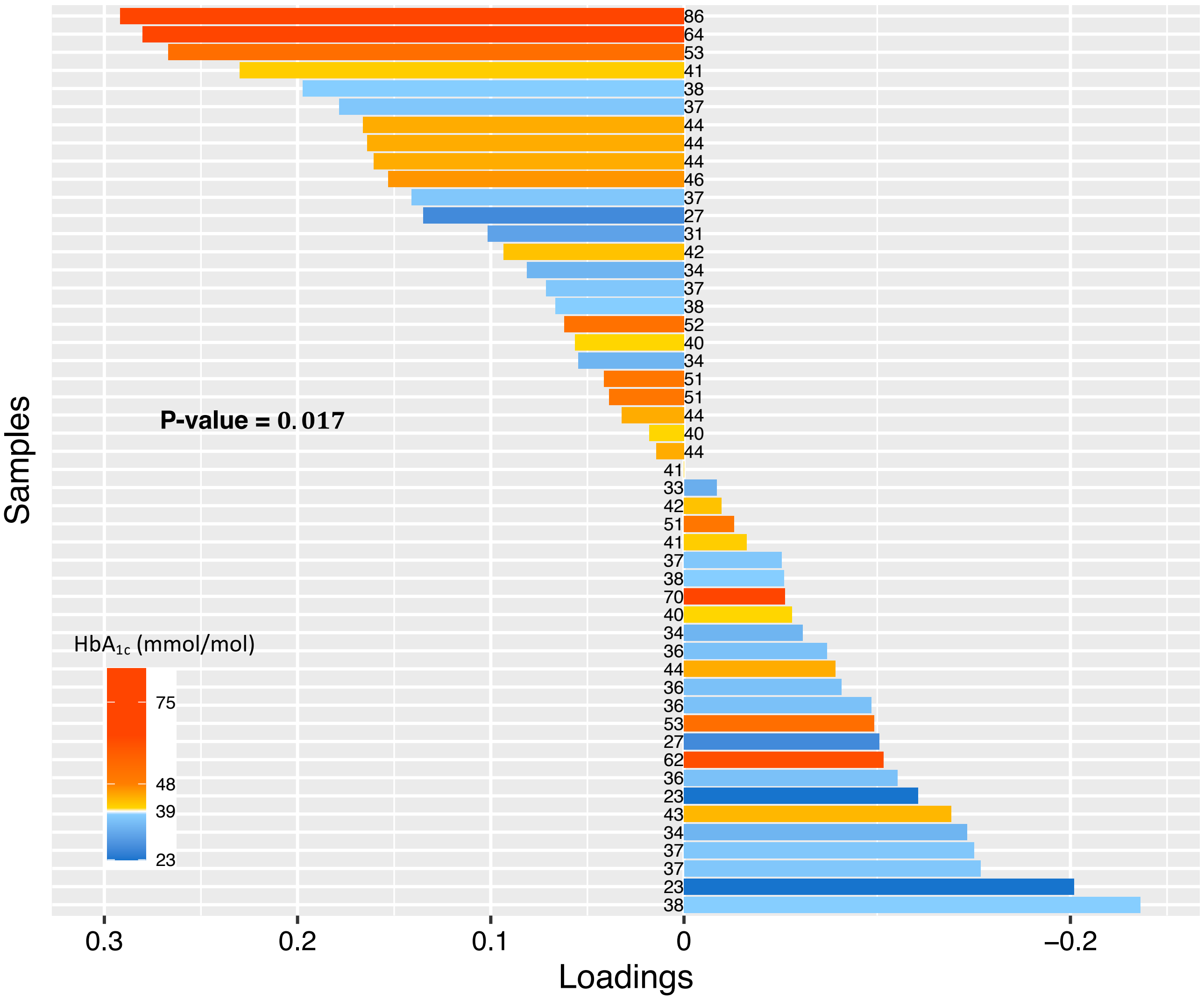

Figure S10. Sorted first principal sample-eigenvector of human. Human samples are displayed in the decreasing order of the first principal sample loadings. Sample HbA_{1c} levels (the digit next to each bar) are indicated by the bar color depth. Normal range for HbA_{1c} level is between 20 mmol/mol (4%) and 38 mmol/mol (5.6%); HbA_{1c} levels between 39 mmol/mol (5.7%) and 46 mmol/mol (6.4%) indicate prediabetes; levels of 48 mmol/mol (6.5%) or higher mean diabetes. The samples with HbA_{1c} levels higher than 39 mmol/mol have overall greater loadings than those with HbA_{1c} levels lower than 39 mmol/mol. The p-value of such a contrast is 0.017, as evaluated by the Wilcoxon rank-sum test.
