# Supplementary material for: A data integration approach unveils a transcriptional signature of type 2 diabetes progression in rat and human islets
Source: PLoS One. 2023 Oct 10;18(10):e0292579. doi: 10.1371/journal.pone.0292579 (PMC10564241; doi:10.1371/journal.pone.0292579)
Supplement: S1 Table — (DOCX) [file pone.0292579.s015.docx]

Table S1. Top three squared singular values of the two pancreatic islet expression profiles and their percentages**.**

| **Singular value ranking** | **Rat** | | | **Human** | | |
| --- | --- | --- | --- | --- | --- | --- |
|  | **Value** | **Percentage** | **Cumulative percentage** | **Value** | **Percentage** | **Cumulative percentage** |
| s1 | 56155.15 | 26.11% | 26.11% | 19950.63 | 18.69% | 18.69% |
| s2 | 26654.92 | 12.39% | 38.50% | 13319.06 | 12.47% | 31.16% |
| s3 | 14605.27 | 6.79% | 49.29% | 11486.65 | 10.76% | 41.92% |
