# Supplementary material for: A data integration approach unveils a transcriptional signature of type 2 diabetes progression in rat and human islets
Source: PLoS One. 2023 Oct 10;18(10):e0292579. doi: 10.1371/journal.pone.0292579 (PMC10564241; doi:10.1371/journal.pone.0292579)
Supplement: S2 Table — (DOCX) [file pone.0292579.s016.docx]

Table S2. Significantly up-regulated genes involved in the angiogenesis-related GO biological processes in the aggregated gene-eigenvector**.**

| **Symbol** | **Rank** | **P-value** | **Gene title** |
| --- | --- | --- | --- |
| *IL6* | 3 | 7.72E-06 | interleukin 6 |
| *PTGS2* | 6 | 2.01E-05 | prostaglandin-endoperoxide synthase 2 |
| *FGF2* | 9 | 7.27E-05 | fibroblast growth factor 2 |
| *C3* | 10 | 8.62E-05 | complement component 3 |
| *THBS2* | 11 | 1.05E-04 | thrombospondin 2 |
| *CFH* | 16 | 1.55E-04 | complement component factor h |
| *DCN* | 21 | 2.20E-04 | decorin |
| *PLAU* | 24 | 2.39E-04 | plasminogen activator, urokinase |
| *SERPINF1* | 30 | 2.93E-04 | serine (or cysteine) peptidase inhibitor, clade F, member 1 |
| *CCL2* | 47 | 5.94E-04 | chemokine (C-C motif) ligand 2 |
| *SLIT2* | 48 | 6.22E-04 | slit homolog 2 (Drosophila) |
| *RUNX1* | 49 | 6.57E-04 | runt related transcription factor 1 |
| *ANXA1* | 61 | 9.26E-04 | annexin A1 |
| *LIF* | 69 | 1.07E-03 | leukemia inhibitory factor |
| *ITGB8* | 80 | 1.40E-03 | integrin beta 8 |
| *MMP2* | 91 | 1.71E-03 | matrix metallopeptidase 2 |
| *APOD* | 94 | 1.79E-03 | apolipoprotein D |
| *THBS1* | 109 | 2.29E-03 | thrombospondin 1 |
| *ADAMTS9* | 111 | 2.32E-03 | a disintegrin-like and metallopeptidase (reprolysin type) with thrombospondin type 1 motif, 9 |
| *TGFBR2* | 115 | 2.43E-03 | transforming growth factor, beta receptor II |
| *TGFB2* | 118 | 2.50E-03 | transforming growth factor, beta 2 |
| *SERPINE1* | 120 | 2.56E-03 | serine (or cysteine) peptidase inhibitor, clade E, member 1 |
| *MIR21* | 143 | 3.21E-03 |  |
| *HK2* | 154 | 3.45E-03 | hexokinase 2 |
| *CLIC4* | 190 | 4.69E-03 | chloride intracellular channel 4 (mitochondrial) |
| *ADAMTS1* | 214 | 5.35E-03 | a disintegrin-like and metallopeptidase (reprolysin type) with thrombospondin type 1 motif, 1 |
| *SPHK1* | 221 | 5.53E-03 | sphingosine kinase 1 |
| *PTPN14* | 229 | 5.79E-03 | protein tyrosine phosphatase, non-receptor type 14 |
| *PTPRM* | 236 | 6.08E-03 | protein tyrosine phosphatase, receptor type, M |
| *CTNNB1* | 242 | 6.41E-03 | catenin (cadherin associated protein), beta 1 |
| *CYP1B1* | 250 | 6.57E-03 | cytochrome P450, family 1, subfamily b, polypeptide 1 |
| *HGF* | 263 | 6.96E-03 | hepatocyte growth factor |
| *MYH9* | 264 | 6.96E-03 | myosin, heavy polypeptide 9, non-muscle |
| *ANXA2* | 267 | 6.98E-03 | annexin A2 |
| *KLF4* | 268 | 7.03E-03 | Kruppel-like factor 4 (gut) |
| *JAK1* | 286 | 7.61E-03 | Janus kinase 1 |
| *JAG1* | 294 | 7.83E-03 | jagged 1 |
| *DDAH1* | 310 | 8.37E-03 | dimethylarginine dimethylaminohydrolase 1 |
| *CXCL8* | 313 | 8.56E-03 | Interleukin-8 |
| *MMP14* | 321 | 8.75E-03 | matrix metallopeptidase 14 (membrane-inserted) |
| *SAT1* | 324 | 8.84E-03 | spermidine/spermine N1-acetyl transferase 1 |
| *SMOC2* | 329 | 8.96E-03 | SPARC related modular calcium binding 2 |
| *ELK3* | 356 | 9.99E-03 | ELK3, member of ETS oncogene family |
| *RHOJ* | 365 | 0.010 | ras homolog gene family, member J |
| *KLF5* | 367 | 0.010 | Kruppel-like factor 5 |
| *CALD1* | 369 | 0.010 | caldesmon 1 |
| *NRP1* | 380 | 0.011 | neuropilin 1 |
| *RNF213* | 381 | 0.011 | ring finger protein 213 |
| *CASP8* | 390 | 0.011 | caspase 8 |
| *CYBB* | 395 | 0.011 | cytochrome b-245, beta polypeptide |
| *COL15A1* | 415 | 0.012 | collagen, type XV, alpha 1 |
| *SEMA6A* | 421 | 0.012 | sema domain, transmembrane domain (TM), and cytoplasmic domain, (semaphorin) 6A |
| *JUN* | 422 | 0.012 | jun proto-oncogene |
| *ANGPTL4* | 424 | 0.012 | angiopoietin-like 4 |
| *EPHB2* | 467 | 0.014 | Eph receptor B2 |
| *FBLN5* | 486 | 0.014 | fibulin 5 |
| *HDAC7* | 496 | 0.015 | histone deacetylase 7 |
| *HSPB1* | 498 | 0.015 | heat shock protein 1 |
| *FLNA* | 499 | 0.015 | filamin, alpha |
| *THBS4* | 503 | 0.015 | thrombospondin 4 |
| *FGFR2* | 518 | 0.015 | fibroblast growth factor receptor 2 |
| *ANXA3* | 520 | 0.016 | annexin A3 |
| *PPP1R15A* | 525 | 0.016 | protein phosphatase 1, regulatory (inhibitor) subunit 15A |
| *B4GALT1* | 534 | 0.016 | UDP-Gal:betaGlcNAc beta 1,4- galactosyltransferase, polypeptide 1 |
| *FGFR1* | 538 | 0.016 | fibroblast growth factor receptor 1 |
| *ZC3H12A* | 553 | 0.017 | zinc finger CCCH type containing 12A |
| *FGFBP1* | 554 | 0.017 | fibroblast growth factor binding protein 1 |
| *ITGAV* | 565 | 0.017 | integrin alpha V |
| *ITGA5* | 569 | 0.017 | integrin alpha 5 (fibronectin receptor alpha) |
| *PDGFRB* | 575 | 0.018 | platelet derived growth factor receptor, beta polypeptide |
| *ADAM12* | 580 | 0.018 | a disintegrin and metallopeptidase domain 12 (meltrin alpha) |
| *PDGFA* | 595 | 0.018 | platelet derived growth factor, alpha |
| *TJP1* | 608 | 0.019 | tight junction protein 1 |
| *SRPX2* | 652 | 0.021 | sushi-repeat-containing protein, X-linked 2 |
| *TNFAIP2* | 656 | 0.021 | tumor necrosis factor, alpha-induced protein 2 |
| *HIF1A* | 657 | 0.021 | hypoxia inducible factor 1, alpha subunit |
| *SFRP1* | 662 | 0.021 | secreted frizzled-related protein 1 |
| *GATA6* | 665 | 0.021 | GATA binding protein 6 |
| *FMNL3* | 666 | 0.021 | formin-like 3 |
| *EPHB3* | 672 | 0.022 | Eph receptor B3 |
| *FGF10* | 697 | 0.023 | fibroblast growth factor 10 |
| *PARVA* | 701 | 0.023 | parvin, alpha |
| *LEMD3* | 714 | 0.024 | LEM domain containing 3 |
| *TNFRSF1A* | 718 | 0.024 | tumor necrosis factor receptor superfamily, member 1a |
| *IL1B* | 721 | 0.025 | interleukin 1 beta |
| *BMPER* | 729 | 0.025 | BMP-binding endothelial regulator |
| *SHB* | 738 | 0.025 | src homology 2 domain-containing transforming protein B |
| *UBP1* | 761 | 0.026 | upstream binding protein 1 |
| *SPRED1* | 779 | 0.027 | sprouty protein with EVH-1 domain 1, related sequence |
| *RSPO3* | 808 | 0.028 | R-spondin 3 |
| *SASH1* | 809 | 0.028 | SAM and SH3 domain containing 1 |
| *LRG1* | 818 | 0.029 | leucine-rich alpha-2-glycoprotein 1 |
| *AGO2* | 826 | 0.029 | argonaute RISC catalytic subunit 2 |
| *EPAS1* | 829 | 0.029 | endothelial PAS domain protein 1 |
| *NRP2* | 850 | 0.030 | neuropilin 2 |
| *NCL* | 857 | 0.030 | nucleolin |
| *CCL11* | 866 | 0.030 | chemokine (C-C motif) ligand 11 |
| *CALCRL* | 891 | 0.031 | calcitonin receptor-like |
| *HIPK2* | 895 | 0.032 | homeodomain interacting protein kinase 2 |
| *AQP1* | 906 | 0.032 | aquaporin 1 |
| *TGFA* | 920 | 0.033 | transforming growth factor alpha |
| *SYK* | 924 | 0.033 | spleen tyrosine kinase |
| *WASF2* | 937 | 0.033 | WAS protein family, member 2 |
| *EGF* | 975 | 0.035 | epidermal growth factor |
| *SPRY2* | 985 | 0.035 | sprouty homolog 2 (Drosophila) |
| *NR4A1* | 987 | 0.035 | nuclear receptor subfamily 4, group A, member 1 |
| *HHEX* | 989 | 0.035 | hematopoietically expressed homeobox |
| *MIR23B* | 992 | 0.036 |  |
| *PDPN* | 1001 | 0.036 | podoplanin |
| *PTPRB* | 1052 | 0.038 | protein tyrosine phosphatase, receptor type, B |
| *VEGFD* | 1081 | 0.039 | vascular endothelial growth factor D |
| *NFE2L2* | 1092 | 0.040 | nuclear factor, erythroid derived 2, like 2 |
| *CXCL10* | 1098 | 0.040 | chemokine (C-X-C motif) ligand 10 |
| *TCF4* | 1105 | 0.040 | transcription factor 4 |
| *FN1* | 1111 | 0.040 | fibronectin 1 |
| *AMOTL1* | 1112 | 0.040 | angiomotin-like 1 |
| *TEK* | 1119 | 0.040 | endothelial-specific receptor tyrosine kinase |
| *LGALS3* | 1145 | 0.041 | lectin, galactose binding, soluble 3 |
| *ANPEP* | 1147 | 0.042 | alanyl (membrane) aminopeptidase |
| *STAT3* | 1169 | 0.042 | signal transducer and activator of transcription 3 |
| *ROCK2* | 1173 | 0.042 | Rho-associated coiled-coil containing protein kinase 2 |
| *EPHB4* | 1200 | 0.044 | Eph receptor B4 |
| *SP100* | 1217 | 0.044 | nuclear antigen Sp100 |
| *ARHGAP22* | 1234 | 0.045 | Rho GTPase activating protein 22 |
| *RBPJ* | 1258 | 0.046 | recombination signal binding protein for immunoglobulin kappa J region |
| *COL4A2* | 1277 | 0.047 | collagen, type IV, alpha 2 |
| *NF1* | 1286 | 0.047 | neurofibromatosis 1 |
| *FGFRL1* | 1295 | 0.048 | fibroblast growth factor receptor-like 1 |
| *GADD45A* | 1310 | 0.048 | growth arrest and DNA-damage-inducible 45 alpha |
| *ADAM8* | 1339 | 0.050 | a disintegrin and metallopeptidase domain 8 |
