# Supplementary material for: A data integration approach unveils a transcriptional signature of type 2 diabetes progression in rat and human islets
Source: PLoS One. 2023 Oct 10;18(10):e0292579. doi: 10.1371/journal.pone.0292579 (PMC10564241; doi:10.1371/journal.pone.0292579)
Supplement: S3 Table — (DOCX) [file pone.0292579.s017.docx]

Table S3. Significantly up-regulated endothelial cell marker genes in the aggregated gene-eigenvector**.**

| **Symbol** | **Rank** | **P-value** | **Gene Title** |
| --- | --- | --- | --- |
| ***VCAM1*** | 4 | 1.53E-05 | vascular cell adhesion molecule 1 |
| ***CD44*** | 27 | 2.61E-04 | CD44 antigen |
| ***CD93*** | 128 | 2.85E-03 | CD93 antigen |
| *SELP* | 187 | 4.64E-03 | selectin, platelet |
| ***NOTCH2*** | 194 | 4.76E-03 | notch 2 |
| *IL1R1* | 197 | 4.82E-03 | interleukin 1 receptor, type I |
| ***DCBLD2*** | 224 | 5.63E-03 | discoidin, CUB and LCCL domain containing 2 |
| ***ICAM1*** | 225 | 5.64E-03 | intercellular adhesion molecule 1 |
| ***KLF4*** | 268 | 7.03E-03 | Kruppel-like factor 4 (gut) |
| ***ADAM9*** | 352 | 9.85E-03 | a disintegrin and metallopeptidase domain 9 (meltrin gamma) |
| ***SELE*** | 387 | 0.0108 | selectin, endothelial cell |
| *PODXL* | 527 | 0.0159 | podocalyxin-like |
| ***ADAM12*** | 580 | 0.0176 | a disintegrin and metallopeptidase domain 12 (meltrin alpha) |
| ***CXCL16*** | 679 | 0.0221 | chemokine (C-X-C motif) ligand 16 |
| ***PDPN*** | 1001 | 0.0361 | podoplanin |
| ***ANTXR1*** | 1071 | 0.0390 | anthrax toxin receptor 1 |
| ***ADAM10*** | 1096 | 0.0397 | a disintegrin and metallopeptidase domain 10 |
| ***LYVE1*** | 1101 | 0.0398 | lymphatic vessel endothelial hyaluronan receptor 1 |
| ***TEK*** | 1119 | 0.0404 | endothelial-specific receptor tyrosine kinase |
| ***ANPEP*** | 1147 | 0.0415 | alanyl (membrane) aminopeptidase |
| *CD86* | 1163 | 0.0420 | CD86 antigen |
| ***VWF*** | 1300 | 0.0479 | Von Willebrand factor homolog |
| ***ADAM8*** | 1339 | 0.0499 | a disintegrin and metallopeptidase domain 8 |

Genes related to angiogenesis are highlighted in bold.
