# Supplementary material for: A data integration approach unveils a transcriptional signature of type 2 diabetes progression in rat and human islets
Source: PLoS One. 2023 Oct 10;18(10):e0292579. doi: 10.1371/journal.pone.0292579 (PMC10564241; doi:10.1371/journal.pone.0292579)
Supplement: S4 Table — (DOCX) [file pone.0292579.s018.docx]

Table S4. Significantly up-regulated genes involved in the KEGG “VEGF signaling pathway” in the aggregated gene-eigenvector**.**

| **Symbol** | **Rank** | **P-value** | **Gene Title** |
| --- | --- | --- | --- |
| *PTGS2* | 6 | 2.0E-05 | prostaglandin-endoperoxide synthase 2 |
| *PLA2G4A* | 90 | 1.7E-03 | phospholipase A2, group IVA (cytosolic, calcium-dependent) |
| *SPHK1* | 221 | 5.5E-03 | sphingosine kinase 1 |
| *PIK3R1* | 289 | 7.7E-03 | phosphatidylinositol 3-kinase, regulatory subunit, polypeptide 1 (p85 alpha) |
| *HSPB1* | 498 | 0.015 | heat shock protein 1 |
| *NFATC2* | 664 | 0.021 | nuclear factor of activated T cells, cytoplasmic, calcineurin dependent 2 |
