# Supplementary material for: A data integration approach unveils a transcriptional signature of type 2 diabetes progression in rat and human islets
Source: PLoS One. 2023 Oct 10;18(10):e0292579. doi: 10.1371/journal.pone.0292579 (PMC10564241; doi:10.1371/journal.pone.0292579)
Supplement: S5 Table — (DOCX) [file pone.0292579.s019.docx]

Table S5. Significantly up-regulated genes involved in the REACTOME pathway “signaling by VEGF” in the aggregated gene-eigenvector**.**

| **Symbol** | **Rank** | **P-value** | **Gene Title** |
| --- | --- | --- | --- |
| *SPHK1* | 221 | 5.53E-03 | sphingosine kinase 1 |
| *CTNNB1* | 242 | 6.41E-03 | catenin (cadherin associated protein), beta 1 |
| *AXL* | 253 | 6.65E-03 | AXL receptor tyrosine kinase |
| *PIK3R1* | 289 | 7.67E-03 | phosphatidylinositol 3-kinase, regulatory subunit, polypeptide 1 (p85 alpha) |
| *NRP1* | 380 | 0.011 | neuropilin 1 |
| *CYBB* | 395 | 0.011 | cytochrome b-245, beta polypeptide |
| *CALM2* | 427 | 0.012 | calmodulin 2 |
| *HSPB1* | 498 | 0.015 | heat shock protein 1 |
| *ITGAV* | 565 | 0.017 | integrin alpha V |
| *SHB* | 738 | 0.025 | src homology 2 domain-containing transforming protein B |
| *NRP2* | 850 | 0.030 | neuropilin 2 |
| *DOCK1* | 872 | 0.031 | dedicator of cytokinesis 1 |
| *WASF2* | 937 | 0.033 | WAS protein family, member 2 |
| *CYFIP1* | 1072 | 0.039 | cytoplasmic FMR1 interacting protein 1 |
| *VEGFD* | 1081 | 0.039 | vascular endothelial growth factor D |
| *CTNND1* | 1138 | 0.041 | catenin (cadherin associated protein), delta 1 |
| *ROCK2* | 1173 | 0.042 | Rho-associated coiled-coil containing protein kinase 2 |
| *PAK1* | 1334 | 0.050 | p21 protein (Cdc42/Rac)-activated kinase 1 |
