# Supplementary material for: A data integration approach unveils a transcriptional signature of type 2 diabetes progression in rat and human islets
Source: PLoS One. 2023 Oct 10;18(10):e0292579. doi: 10.1371/journal.pone.0292579 (PMC10564241; doi:10.1371/journal.pone.0292579)
Supplement: S6 Table — (DOCX) [file pone.0292579.s020.docx]

Table S6. Significantly up-regulated genes involved in the KEGG “HIF-1 signaling pathway” in the aggregated gene-eigenvector**.**

| **Symbol** | **Rank** | **P-value** | **Gene Title** |
| --- | --- | --- | --- |
| *IL6* | 3 | 7.72E-06 | interleukin 6 |
| *EFEMP1* | 36 | 4.26E-04 | epidermal growth factor-containing fibulin-like extracellular matrix protein 1 |
| *PFKFB3* | 85 | 1.52E-03 | 6-phosphofructo-2-kinase/fructose-2,6-biphosphatase 3 |
| *SERPINE1* | 120 | 2.56E-03 | serine (or cysteine) peptidase inhibitor, clade E, member 1 |
| *ALDOB* | 151 | 3.39E-03 | aldolase B, fructose-bisphosphate |
| *HK2* | 154 | 3.45E-03 | hexokinase 2 |
| *NFKB1* | 205 | 5.11E-03 | nuclear factor of kappa light polypeptide gene enhancer in B cells 1, p105 |
| *LDHA* | 254 | 6.67E-03 | lactate dehydrogenase A |
| *INSR* | 260 | 6.84E-03 | insulin receptor |
| *EGFR* | 285 | 7.53E-03 | epidermal growth factor receptor |
| *PIK3R1* | 289 | 7.67E-03 | phosphatidylinositol 3-kinase, regulatory subunit, polypeptide 1 (p85 alpha) |
| *EGR1* | 290 | 7.67E-03 | early growth response 1 |
| *CYBB* | 395 | 0.011 | cytochrome b-245, beta polypeptide |
| *ANGPTL4* | 424 | 0.012 | angiopoietin-like 4 |
| *HK1* | 431 | 0.012 | hexokinase 1 |
| *HIF1A* | 657 | 0.021 | hypoxia inducible factor 1, alpha subunit |
| *TF* | 835 | 0.029 | tissue factor |
| *EGF* | 975 | 0.035 | epidermal growth factor |
| *SERPINE2* | 994 | 0.036 | serine (or cysteine) peptidase inhibitor, clade E, member 2 |
| *TEK* | 1119 | 0.040 | endothelial-specific receptor tyrosine kinase |
| *STAT3* | 1169 | 0.042 | signal transducer and activator of transcription 3 |
| *MKNK2* | 1244 | 0.045 | MAP kinase-interacting serine/threonine kinase 2 |
| *BCL2* | 1266 | 0.046 | B cell leukemia/lymphoma 2 |
