# Supplementary material for: A data integration approach unveils a transcriptional signature of type 2 diabetes progression in rat and human islets
Source: PLoS One. 2023 Oct 10;18(10):e0292579. doi: 10.1371/journal.pone.0292579 (PMC10564241; doi:10.1371/journal.pone.0292579)
Supplement: S7 Table — (DOCX) [file pone.0292579.s021.docx]

Table S7. Significantly up-regulated genes related to hypoxia in the aggregated gene-eigenvector**.**

| **Symbol** | **Rank** | **P-value** | **Gene Title** |
| --- | --- | --- | --- |
| *VCAM1* | 4 | 1.53E-05 | vascular cell adhesion molecule 1 |
| *PLAU* | 24 | 2.39E-04 | plasminogen activator, urokinase |
| *CCL2* | 47 | 5.94E-04 | chemokine (C-C motif) ligand 2 |
| *BMP2* | 65 | 1.02E-03 | bone morphogenetic protein 2 |
| *DIO3* | 82 | 1.43E-03 | Thyroxine 5-deiodinase |
| *MMP2* | 91 | 1.71E-03 | matrix metallopeptidase 2 |
| *REST* | 107 | 2.20E-03 | RE1-silencing transcription factor |
| *THBS1* | 109 | 2.29E-03 | thrombospondin 1 |
| *TGFBR2* | 115 | 2.43E-03 | transforming growth factor, beta receptor II |
| *TGFB2* | 118 | 2.50E-03 | transforming growth factor, beta 2 |
| *TGFB3* | 119 | 2.51E-03 | transforming growth factor, beta 3 |
| *ITGA2* | 124 | 2.68E-03 | integrin alpha 2 |
| *MIR21* | 143 | 3.21E-03 | microRNA 21 |
| *FOSL2* | 148 | 3.27E-03 | fos-like antigen 2 |
| *PLOD2* | 149 | 3.31E-03 | procollagen lysine, 2-oxoglutarate 5-dioxygenase 2 |
| *HK2* | 154 | 3.45E-03 | hexokinase 2 |
| *MMP3* | 164 | 3.75E-03 | matrix metallopeptidase 3 |
| *ICAM1* | 225 | 5.64E-03 | intercellular adhesion molecule 1 |
| *SMAD3* | 247 | 6.49E-03 | SMAD family member 3 |
| *LDHA* | 254 | 6.67E-03 | lactate dehydrogenase A |
| *INSR* | 260 | 6.84E-03 | insulin receptor |
| *EGR1* | 290 | 7.67E-03 | early growth response 1 |
| *MMP14* | 321 | 8.75E-03 | matrix metallopeptidase 14 (membrane-inserted) |
| *REG1A* | 335 | 9.28E-03 | Lithostathine-1-alpha |
| *CD24* | 359 | 0.010 | Signal transducer CD24 |
| *CXCL12* | 370 | 0.010 | chemokine (C-X-C motif) ligand 12 |
| *MUC1* | 378 | 0.011 | mucin 1, transmembrane |
| *ANGPTL4* | 424 | 0.012 | angiopoietin-like 4 |
| *HK1* | 431 | 0.012 | hexokinase 1 |
| *EDNRA* | 435 | 0.012 | endothelin receptor type A |
| *DDIT4* | 506 | 0.015 | DNA-damage-inducible transcript 4 |
| *POSTN* | 555 | 0.017 | periostin, osteoblast specific factor |
| *SOX4* | 561 | 0.017 | SRY (sex determining region Y)-box 4 |
| *NOX4* | 587 | 0.018 | NADPH oxidase 4 |
| *PDGFA* | 595 | 0.018 | platelet derived growth factor, alpha |
| *HIF1A* | 657 | 0.021 | hypoxia inducible factor 1, alpha subunit |
| *CXCR4* | 708 | 0.024 | chemokine (C-X-C motif) receptor 4 |
| *TNFRSF1A* | 718 | 0.024 | tumor necrosis factor receptor superfamily, member 1a |
| *IL1B* | 721 | 0.025 | interleukin 1 beta |
| *PLAT* | 739 | 0.025 | plasminogen activator, tissue |
| *EPAS1* | 829 | 0.029 | endothelial PAS domain protein 1 |
| *TF* | 835 | 0.029 | Tissue factor |
| *TGFB1* | 868 | 0.031 | transforming growth factor, beta 1 |
| *NR4A2* | 878 | 0.031 | nuclear receptor subfamily 4, group A, member 2 |
| *MMP12* | 893 | 0.031 | matrix metallopeptidase 12 |
| *PPARGC1A* | 1014 | 0.036 | peroxisome proliferative activated receptor, gamma, coactivator 1 alpha |
| *APAF1* | 1024 | 0.037 | apoptotic peptidase activating factor 1 |
| *BCL2L1* | 1047 | 0.038 | BCL2-like 1 |
| *CRYAB* | 1076 | 0.039 | crystallin, alpha B |
| *VEGFD* | 1081 | 0.039 | Vascular endothelial growth factor D |
| *CFLAR* | 1109 | 0.040 | CASP8 and FADD-like apoptosis regulator |
| *TEK* | 1119 | 0.040 | endothelial-specific receptor tyrosine kinase |
| *PENK* | 1188 | 0.043 | preproenkephalin |
| *BCL2* | 1266 | 0.046 | B cell leukemia/lymphoma 2 |
| *BIRC2* | 1280 | 0.047 | baculoviral IAP repeat-containing 2 |
| *NF1* | 1286 | 0.047 | neurofibromatosis 1 |
| *PAK1* | 1334 | 0.050 | p21 protein (Cdc42/Rac)-activated kinase 1 |
