# Supplementary material for: A data integration approach unveils a transcriptional signature of type 2 diabetes progression in rat and human islets
Source: PLoS One. 2023 Oct 10;18(10):e0292579. doi: 10.1371/journal.pone.0292579 (PMC10564241; doi:10.1371/journal.pone.0292579)
Supplement: S8 Table — (DOCX) [file pone.0292579.s022.docx]

Table S8. Inflammation-related pathways enriched at the positive poles of the angiogenesis gene-eigenvectors of the two species**.**

| **Pathway** | **Database** | **P-value** | |
| --- | --- | --- | --- |
|  |  | **Rat 1^st^** | **Human 2^nd^** |
| Chronic inflammatory response | GO.bp | **1.53E-03** | **1.01E-03** |
| Inflammatory response | GO.bp | **1.43E-23** | **5.50E-15** |
| Inflammatory response to antigenic stimulus | GO.bp | **3.33E-03** | **0.044** |
| Leukocyte migration involved in inflammatory response | GO.bp | **5.38E-03** | 0.490 |
| NOD-like receptor signaling pathway | KEGG | **2.71E-07** | **2.16E-05** |
| TNF signaling pathway | KEGG | **8.28E-09** | **1.28E-11** |
| NF-kappa B signaling pathway | KEGG | **2.54E-08** | **4.55E-10** |
| NF-kB is activated and signals survival | Reactome | **0.021** | 0.065 |
| Positive regulation of cytokine production involved in inflammatory response | GO.bp | **2.19E-04** | **3.56E-03** |
| Positive regulation of I-kappaB kinase/NF-kappaB signaling | GO.bp | **4.44E-07** | **3.74E-06** |
| Positive regulation of inflammatory response | GO.bp | **1.82E-08** | **3.64E-04** |
| Positive regulation of NIK/NF-kappaB signaling | GO.bp | **5.04E-05** | **0.011** |
| Regulation of inflammatory response | GO.bp | **4.78E-03** | **0.014** |

Significant p-values (<0.05) are highlighted in bold.
