# Supplementary material for: A data integration approach unveils a transcriptional signature of type 2 diabetes progression in rat and human islets
Source: PLoS One. 2023 Oct 10;18(10):e0292579. doi: 10.1371/journal.pone.0292579 (PMC10564241; doi:10.1371/journal.pone.0292579)
Supplement: S9 Table — (DOCX) [file pone.0292579.s023.docx]

Table S9. Immune response-related pathways enriched at the positive poles of the angiogenesis gene-eigenvectors of the two species**.**

| **Pathway** | **Database** | **P-value** | |
| --- | --- | --- | --- |
|  |  | **Rat 1^st^** | **Human 2^nd^** |
| Activation of innate immune response | GO.bp | 0.362 | **1.26E-04** |
| Adaptive immune response | GO.bp | **1.42E-06** | **7.35E-04** |
| Adaptive immune system | Reactome | 0.057 | 0.753 |
| B cell receptor signaling pathway | KEGG | **4.49E-04** | **3.49E-03** |
| Complement activation | GO.bp | **9.87E-05** | **0.015** |
| Complement and coagulation cascades | KEGG | **3.96E-11** | **3.81E-04** |
| Complement cascade | Reactome | **1.12E-03** | **0.038** |
| Cytokine signaling in immune system | Reactome | **2.30E-05** | **3.23E-15** |
| Humoral immune response | GO.bp | **9.76E-03** | **0.012** |
| Immune response | GO.bp | **5.54E-18** | **8.73E-10** |
| Immune system | Reactome | **5.02E-09** | **1.55E-06** |
| Immune system process | GO.bp | **5.32E-04** | 0.181 |
| Immunoglobulin mediated immune response | GO.bp | **5.64E-04** | **1.88E-03** |
| Immunoglobulin production involved in immunoglobulin-mediated immune response | GO.bp | **2.96E-03** | 0.424 |
| Innate immune response | GO.bp | **2.08E-09** | **5.34E-11** |
| Innate immune system | Reactome | **6.75E-06** | **0.037** |
| Macrophage activation involved in immune response | GO.bp | **1.97E-03** | **0.044** |
| Macrophage chemotaxis | GO.bp | **0.030** | **0.014** |
| Positive regulation of B cell proliferation | GO.bp | **5.30E-06** | 0.117 |
| Positive regulation of cytokine production involved in immune response | GO.bp | **0.027** | 0.317 |
| Positive regulation of immune response | GO.bp | - | **0.033** |
| Positive regulation of macrophage activation | GO.bp | **8.30E-06** | **1.25E-04** |
| Positive regulation of macrophage chemotaxis | GO.bp | **1.42E-05** | 0.096 |
| Positive regulation of T cell differentiation | GO.bp | **1.01E-04** | 0.191 |
| Regulation of adaptive immune response | GO.bp | **0.012** | 0.070 |
| Regulation of innate immune response | GO.bp | **0.014** | **5.32E-03** |
| T cell receptor signaling pathway | KEGG | **4.44E-03** | **3.67E-03** |

Significant p-values (<0.05) are highlighted in bold.
