# Supplementary material for: A data integration approach unveils a transcriptional signature of type 2 diabetes progression in rat and human islets
Source: PLoS One. 2023 Oct 10;18(10):e0292579. doi: 10.1371/journal.pone.0292579 (PMC10564241; doi:10.1371/journal.pone.0292579)
Supplement: S10 Table — (DOCX) [file pone.0292579.s024.docx]

Table S10. P-values of angiogenesis-related pathways evidenced by enrichment analysis of human’s first principal gene-eigenvector**.**

| **Pathway** | **Database** | **P-value** | **Pole** |
| --- | --- | --- | --- |
| Blood vessel remodeling | GO.bp | 0.307 | - |
| Blood vessel development | GO.bp | **1.08E-03** | - |
| Blood vessel morphogenesis | GO.bp | **0.028** | - |
| Angiogenesis | GO.bp | **0.049** | - |
| Positive regulation of angiogenesis | GO.bp | 0.062 | - |
| Vasculogenesis | GO.bp | 0.174 | - |
| Positive regulation of VEGF receptor signaling pathway | GO.bp | 0.525 | - |
| VEGF receptor signaling pathway | GO.bp | **0.021** | - |
| Positive regulation of VEGF production | GO.bp | **0.033** | - |
| VEGF production | GO.bp | 0.492 | - |
| Signaling by VEGF | Reactome | **2.60E-03** | - |
| VEGF signaling pathway | KEGG | 0.069 | - |
| VEGFA-VEGFR2 Pathway | Reactome | **9.89E-03** | - |
| Sprouting angiogenesis | GO.bp | **2.89E-03** | - |

Significant p-values (<0.05) are highlighted in bold. 8/14 of the listed angiogenesis-related pathways are enriched at the negative pole of human’s first principal gene-eigenvector (a rather late stage of type 2 diabetes) and the remaining pathways are not significant.
